# Characteristics and differences in immune response capacity and gut microbiome between wild and captive Amur grayling (*Thymallus grubii*): New insights into endangered fish conservation

**DOI:** 10.3389/fimmu.2025.1654437

**Published:** 2025-11-19

**Authors:** Cunhua Zhai, Ziyang Wang, Luye Bai, Haoxiang Han, Bo Ma

**Affiliations:** 1 Heilongjiang River Fishery Research Institute, Chinese Academy of Fishery Sciences, Harbin, China; 2 Research Station for Wild Scientific Observation on Fishery Resources and Ecological Environment Protection, Jiamusi, Ministry of Agriculture and Rural Affairs, Jiamusi, China; 3 Scientific Observation Station of Fisheries Resource and Environment in Heilongjiang River Basin, Ministry of Agriculture and Rural Affairs, Harbin, China; 4 College of Life Science and Technology, Harbin Normal University, Harbin, China; 5 College of Fisheries and Life Science, Dalian Ocean University, Dalian, China

**Keywords:** *Thymallus grubii*, liver, gut microbiota, immunity, conservation biology

## Abstract

**Introduction:**

Endangered species recovery hinges on evaluating captivity-induced shifts in the adaptive traits of candidates slated for reintroduction. Gut microbiota is one such trait and is particularly important for Amur grayling (*Thymallus grubii*).

**Methods:**

The present study aimed to systematically investigate the differences in intestinal and liver health in Amur grayling from two water environments (wild and captive) by feeding habits, biochemical parameters and 16S ribosomal RNA gene sequencing.

**Results:**

Compared with captive fish, the wild Amur grayling in the liver and gut had higher lysozyme activity (*P* < 0.05), and alkaline phosphatase, catalase activity and glutathione content in gut was significantly higher (*P* < 0.05). In addition, the cultured fish showed lower relative expression levels of hepatic *IgM, il-6, il-10, il-lβ, myd88, NF-kB*, and *Tnf-α* mRNA expressions than those of wild fish (*P* < 0.05). In the intestine tissue, the mRNA level of *C3, il-6, il-10, il-lβ, tlr1, tlr3, Tnf-α*, and LYZ increased in the wild fish while the expression of *IgM* was significantly elevated (*P* < 0.05). For gut microbiota, the cultured group displayed higher percentages of Pseudomonadota phylum and lower percentages of Bacillota phylum than the wild group (*P* < 0.05) .

**Discussion:**

Overall, wild Amur grayling had higher immune capacity and intestinal barrier functions than cultured Amur grayling. This study displayed that responses and adaptations to diverse aquatic environments were shown by liver-gut-microbiota axis in Amur grayling. Our findings could provide a promising direction for the improve its adaptability of wild population in reintroduction project and propose the conservation strategy for biodiversity recovery.

## 1 Introduction

Amur grayling (*Thymallus grubii*) is a cold-water freshwater fish species of high nutritional value in China, but it currently faces numerous threats caused by anthropogenic activities, including environmental pollution and overfishing (1). The species has been classified as protected and listed in the “China Red Data Book of Endangered Animals” (2). Consequently, captive breeding in artificially controlled environments is widely used to conserve the limited Amur grayling population in China. However, many attempts to reintroduce the endangered species into its natural habitat have not been successful. One potential reason is the loss of adaptive traits (3), such as alterations in host-associated microbiome composition.

Gut microbiota can be regarded as a signaling hub that integrates environmental inputs with host immune capacity (4, 5). In aquatic animals, wild and captive environments differ significantly in diet, social structure, and sources of stress, which may affect the gut microbial community (6). Differences in gut microbiota composition between wild and hatchery-reared juveniles suggest that the physiological status of juveniles may be influenced by their microbiota (7), potentially conferring a disadvantage after release into the wild. Currently, animal microbiome research has been applied as a perspective for population conservation practices (8). Thus, studies on the gut microbiota in wild and captive animals may provide insights into the adaptation mechanisms of Amur grayling in the wild, which could help increase survival rates during the reintroduction period.

It has been demonstrated that the fish gut microbiome can have significant impacts on host metabolism, immunity, and responses to infection (9, 10). The intestinal microbiota can regulate systemic immunity via signals transmitted through immune cells in the intestine (11). Notably, the liver is anatomically connected to the gut through the portal venous circulation, commonly referred to as the “gut–liver axis”, thereby establishing a close physiological relationship between gut and liver tissues (12). Several studies have shown that the gut microbiota, by coevolving with the host in a mutualism system, coordinates the host’s immunity, metabolism, barrier protection, and structural functions (13, 14). In turn, the host immune system contributes to tolerating beneficial bacteria taxa while inhibiting the growth of harmful bacteria (15). Some studies have reported relationships between microbes and host immune function to compare the health of animals living in farmed and wild environments, such as ricefield eel (*Monopterus albus*) (16, 17) and pike perch (*Sander Lucioperca*) (18). However, no prior research has compared the effects of captivity and wild conditions on the physiological functions, metabolism, and immune capacities of Amur grayling in an integrated manner. Such information is essential for improving conditions for captive individuals, thereby aiding in Amur grayling population restoration.

This study aimed to compare immune enzyme activities, immune-related gene expression, and gut microbiota composition between wild and captive Amur grayling, and to identify potential probiotic taxa for conservation and aquaculture enhancement. It also explored the possible relationships among adaptations and immunoregulatory responses of Amur grayling under wild and captive environments from the perspective of gut microbiota–immune interaction.

## 2 Materials and methods

### 2.1 Animal ethics statement

All animal experimental procedures were conducted in accordance with the guidelines of the Laboratory Animal Ethics Committee of the Research Institute of Fisheries of the Heilongjiang River (No. 20240909-001), and the guidelines of EU Directive 2010/63/EU for animal experiments were followed throughout the study.

### 2.2 Fish sampling

Cultured Amur grayling (CAG; n = 15; body length : 19.63 cm ± 0.92 cm; body weight: 112.98 g ± 24.89 g) were obtained from the Bohai Cold Water Fish Experimental Station of the Heilongjiang Institute of Fisheries Research, China. Wild Amur grayling (WAG; n = 15; body length: 18.95 cm ± 2.06 cm; body weight: 108.64 g ± 15.75 g) were captured in the Huma River, part of the upper Amur River, in October 2024. CAG came from the same cohort fed with commercial pellets, and the feed nutrient contents are presented in **Supplementary Table S1**. Fish were anesthetized with 100 mg/L tricaine methanesulfonate (MS-222, Sigma, Michigan, USA), after which the stomach, intestine, liver, and intestinal contents were immediately collected and stored in RNase-free tubes for subsequent analysis of feeding habits, biochemical measurements, quantitative real-time PCR (qRT-PCR), and gut microbiome analysis. All samples were immediately frozen in liquid nitrogen for 2 h and then stored at − 80°C.

### 2.3 Observation of feeding habits

The feeding habits of the Amur grayling were assessed by dissecting the fish and removing its stomach. The stomach was then immediately opened, and its contents were extracted and placed on a Petri dish containing 5 mL of sterile physiological saline solution as a diluent. Stomach contents that could be observed directly were separated by type. The frequency of occurrence for each food type was recorded, and its volume was measured. Observation of the stomach contents was performed using a digital microscope (S6D, Leica, Weztlar, Germany) at a magnification of 40 × 10 to determine the types of food consumed by the fish. Contents that could not be identified based on external characteristics due to gastric digestion were verified by DNA extraction, amplification, and sequencing. Ultimately, the contents were identified by aligning the sequencing data against reference sequences in the GenBank database (Release 262.0, 15 August 2024).

### 2.4 Enzyme activity measurements

The activities of acid phosphatase (ACP, JL-T1094), alkaline phosphatase (AKP, JL-T0946), lysozyme (LYZ, JL-T1062), superoxide dismutase (SOD, JL-T0779), catalase (CAT, JL-T0900), glutathione (GSH, JL-T0906), as well as the content of malondialdehyde (MDA, JL-T0761) in the gut and liver, were measured using commercial test kits (Jianglai Co. Ltd., Shanghai, China) following the manufacturer’s instructions. Liver and gut samples were homogenized with cold extraction solution at a ratio of 1:9 (*w:v*, 1 g tissue per 9 mL ice-cold extraction buffer). Following centrifugation at 12,000 rpm for 10 min at 4 °C, the supernatant was collected (19). Optical density (OD) values were measured using the absorbance microplate reader (SpectraMax Plus 384, Molecular Devices, California, USA) at 405 nm (AKP), 405 nm (ACP), 530 nm (LZM), 560 nm (SOD), 510 nm (CAT), 412 nm (GSH), and 532/600 nm (MDA), respectively. Finally, the fresh weight of samples was used to calculate enzymatic activity according to the formula.

### 2.5 RNA extraction, cDNA synthesis, and real-time polymerase chain reaction

Primers were designed using the Primer Premier 6.0 software based on immune- and inflammatory-related gene sequences published in GenBank (**Table 1**). Primer amplification efficiency and linearity were rigorously assessed via the slope (m) and coefficient of determination (*R*²) of the standard curve; only primer sets exhibiting a slope between − 3.6 and − 3.1 (corresponding to 90%–110% efficiency) and an *R*^2^ > 0.98 were advanced to subsequent analyses. Specificity was verified by a single, distinct melt-curve peak and the exclusive presence of a single, target-sized amplicon on agarose gel electrophoresis.

**Table 1:** Primer sequences used for qRT-PCR.

Gene	Primer sequence	Product length (bp)	Accession number
*C3*	F: TGTCTGAGGGTGTGCTGATTC R: TGTCTGGAACCCGATCAACTG	117	XM_042764266.1
*foxo1*	F: TGTGGCCTGATTCCCTTGAC R: TGGGGACTGTGGTTGTGATG	146	XM_021564372.2
*IgM*	F: GCAAATACCCACAGTTCCGC R: GACAAACACCGAAGCACCTG	93	NM_000074.3
*il-6*	F: GCGCTCGTGGTGTTAGTTAAG R: ATCACTTTCTCCCACTTCGGG	134	NM_001124657.1
*il-10*	F: GCTCTCTCCTCCTGTCCCT R: ATGGTGGAGAAGGCGGTG	148	NM_001245099.1
*il-iβ*	F: CCCTGGAGTCTGCCCATTAC R: GAATGTGGTGTTGCGGTTGA	113	XM_021622166.2
*lyz*	F: TCCTCGTGTGAAAGCAAGACA R: GAATCCCTCAAATCCATCAAGCC	84	NM_139180.1
*mpo*	F: CCCTCATCCAACCCTTCATGT R: TTACCTTCCAGCACGACCC	118	NM_000250.2
*myd88*	F: GGATTGCCAGGACCCAACA R: CACAACGTCCTTTCTGTCCAC	115	XM_036943663.1
*NF-κB*	F: GGTGGAAGAGATTTGGGGCA R: ATCATACATGGAAGGGTGGGAG	151	KU238083
*ssa1*	F:CGATGCCAGAGAGAATATCCAGA R: GTCGGAAGTGATTGGGGTCTT	113	NM_199161.5
*tlr1*	F: CTTGGTAGCCAGTTACGTGGT R: TCAGGTGCTTCCAGGTGATG	101	NM_001166101.1
*tlr3*	F: GGAACATAGGTGGAGAATGGGT R: ACTGAGAGGTGAGCTTGCTG	85	NM_001013269.3
*Tnf-α*	F: ACGGTGATGCTGAGTCCAAA R: TCAGTCCACAGTTTGTCCCC	97	NM_001124357.1

Total RNA was extracted from the liver and gut samples using the TRIzol reagent (Thermo Fisher Scientific, Wilmington, DE, USA) according to the manufacturer’s protocol. RNA quality (purity and concentration) was assessed using a NanoDrop ND-1000 spectrophotometer (Thermo Fisher Scientific). RNA degradation and contamination were evaluated by 1% agarose gel electrophoresis. First-strand complementary DNA (cDNA) was synthesized using the Prime Script™ RT reagent kit with DNA Eraser (TaKaRa, Kyoto, Japan). The messenger RNA (mRNA) expression levels of *il-lβ*, *il-6*, *il-10*, *Tnf-α*, *tlr1*, *tlr3*, *myd88*, *NF-κB*, *foxo1*, *igM*, *lyz*, *c3*, *mpo*, and *saa1* were detected by qRT-PCR. The reaction mixture contained 0.4 μL of each forward and reverse primer, 5 μL of 2 × TB Green Premix Ex Taq II (Tli RNaseH Plus), 3 μL of sterile distilled H_2_O (dH_2_O), 0.2 μL of 50 × ROX Reference Dye II (Basel, Roche, Switzerland), and 1 μL of template cDNA. The cycling parameters were established at 95°C for 180 s, followed by 40 cycles of 95°C for 5 s, 60°C for 15 s, and 72°C for 30 s. qRT-PCR was performed using an ABI 7500 real-time PCR instrument (Thermo Fisher Scientific). The level of gene expression was determined using the 2^−ΔΔCT^ method. *β-actin* (F: 5′-GGACTTTGAGCAGGAGATGG-3′; R: 5′-ATGATGGAGTTGTAGGTGGTCT-3′) was used as an internal reference to measure the relative expression of the target genes. All samples were amplified in triplicate (20).

### 2.6 16S ribosomal DNA sequencing and gut microbiome analysis

Three gut content samples from CAG (*n* = 3) and gut content samples from WAG (*n* = 3) were collected from each experimental group. Subsequently, DNA was extracted using the E.Z.N.A. Soil DNA Kit (Omega Bio-tek Inc., Norcross, USA). The purity and integrity of nucleic acids were verified using a NanoDrop 2000 spectrophotometer (Thermo Scientific Inc., USA). The 16S rDNA was then amplified using primers 338F (5′-ACTCCTACGGGAGGCAGCAG-3′) and 806R (5′-GGACTACHVGGGTWTCTAAT-3′) on a thermocycler PCR system (GeneAmp 9700, ABI, California USA) (21). MiSeq library construction and sequencing were performed using the Illumina MiSeq PE300 platform (Illumina, San Diego, California, CA, USA) with a read length of 2 × 300 bp. Raw data underwent quality control and chimera filtering, and operational taxonomic units (OTUs) were obtained using the Uparse algorithm in Vsearch (v2.7.1) clustering. Representative OTU sequences were classified using a native Bayesian model, and species composition was analyzed at the phylum, family, and genus levels. A Venn diagram was used to identify unique and shared OTUs between the CAG and WAG groups. Alpha diversity analyses were performed using QIME software (v1.8.0) to calculate Chao1, Shannon, and Simpson indices. Based on OTUs and species abundance tables, the Bray–Curtis algorithm was used to evaluate the beta diversity of all samples, including unweighted Unifrac distance-based principal coordinate analysis (PCoA) and partial least squares discrimination analysis (PLS-DA). Functional prediction of OTUs and KEGG pathway analysis were performed using Phylogenetic Investigation of Communities by Reconstruction of Unobserved States (PICRUSt2) (v2.2.0). Biomarker features in each group were identified using linear discriminant analysis effect size (LefSe) software (v1.0.0). All bioinformatics data were analyzed using the Majorbio cloud platform (https://www.majorbio.com).

### 2.7 Statistical analysis

The biochemical data were calculated using Microsoft Excel and analyzed using the statistical software package IBM SPSS 22.0 (IBM Corporation, Armonk, NY, USA). All data are presented as the mean ± standard deviation (SD). Prior to statistical analysis, normality and homogeneity of variance were assessed using the Kolmogorov–Smirnov and Levene’s test, respectively. Independent *t*-tests were then utilized to determine the significance of differences, with *p* < 0.05 indicating a significant difference between the two groups. *p*-values from Spearman correlations were adjusted using the Benjamini–Hochberg false-discovery rate (FDR) method, and only correlations with FDR < 0.05 were considered significant.

## 3 Results

### 3.1 Authentication of feeding habits

The results showed that wild Amur grayling was dominated by zoobenthos (97.206%), identified based on morphological characteristics and genetic analysis. Among them, the most dominant diet was Perlidae (37.692%), followed by *Ephemera* (28.200%), *Nothopsyche* (14.002%), and *Eubasilissa* (5.652%). Therefore, the Amur grayling is a carnivorous fish that primarily feeds on zoobenthos. We hypothesize that this diet reflects a match between the fish’s trophic spectrum and locally available prey, thereby minimizing foraging costs. In addition, the abundance of zoobenthos precisely aligns with the realized nutritional niche of Amur grayling.

### 3.2 Analysis of immune enzyme activities

**Figure 1:**
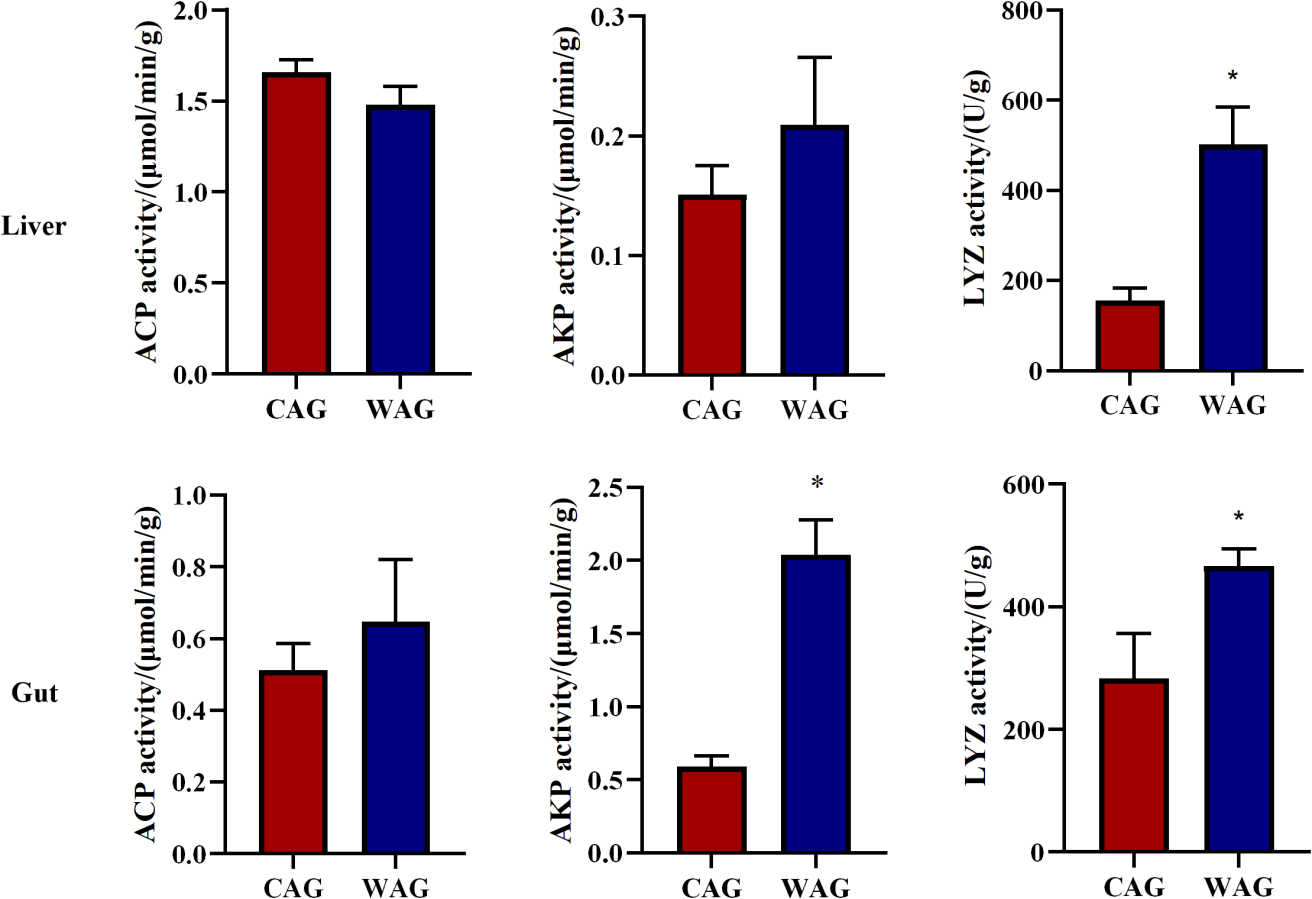
Intestinal and haptic immune-related enzymes of wild (WAG) and cultured (CAG) Amur grayling. Results are shown as mean ± SD (*n* = 5). Values marked with asterisks are significantly different (^*^p < 0.05).

Changes in ACP, AKP, and LYZ levels in liver and gut tissue are summarized in **Figure 1**. The activities of ACP in the liver and gut showed no significant differences (*p* > 0.05) between the WAG and CAG groups. AKP activity in the gut was significantly higher in the WAG group than in the CAG group (*p* < 0.05), while AKP activity in the liver showed no significant difference between the two groups (*p* > 0.05). Similarly, LYZ activity in the liver and gut tissues of Amur grayling was significantly higher in the WAG group than in the CAG group (*p* < 0.05), demonstrating that the wild environment can enhance immunity in Amur grayling.

### 3.3 Analysis of antioxidant parameters

**Figure 2:**
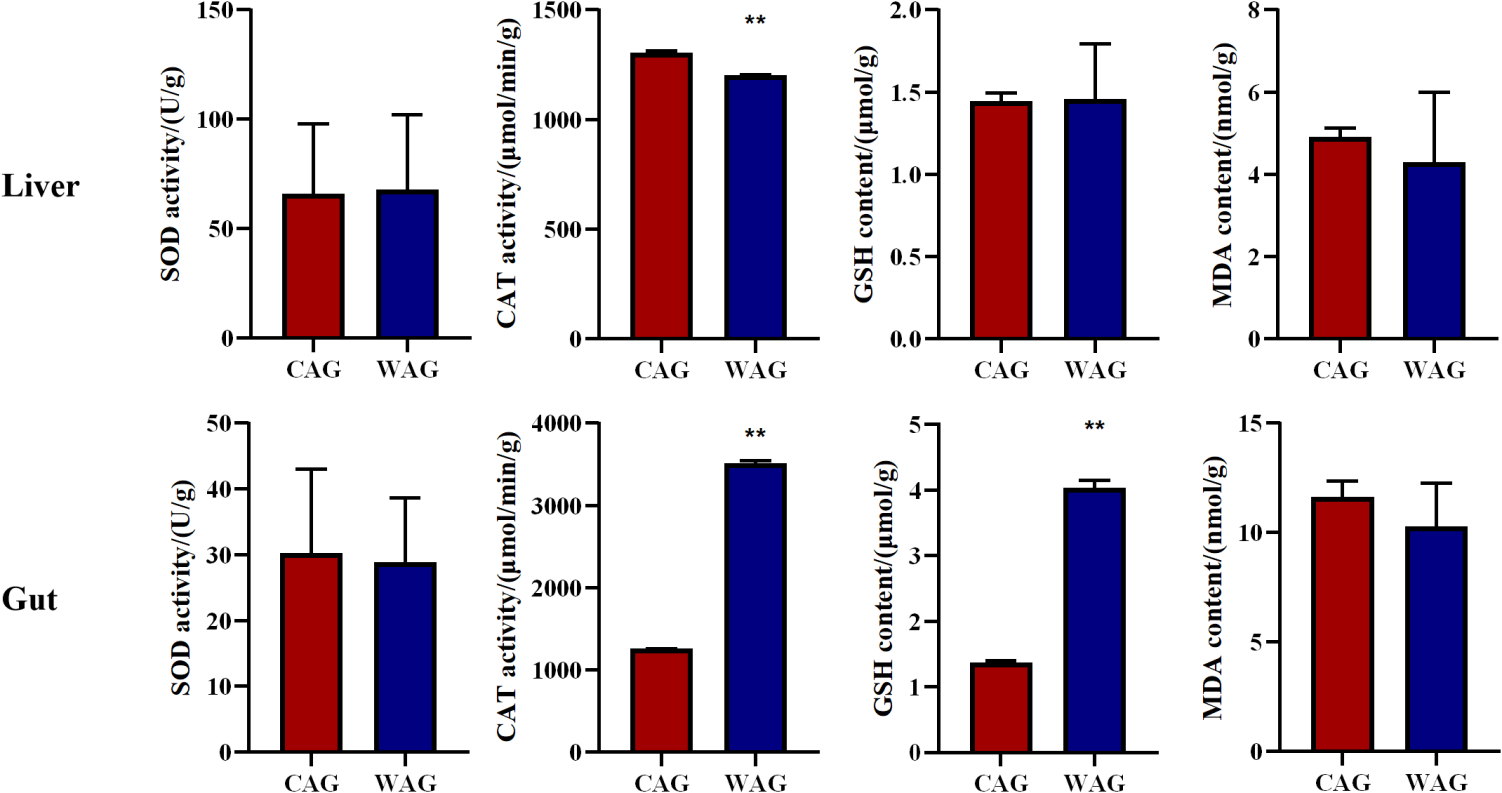
Intestinal and haptic antioxidant-related parameters of wild (WAG) and cultured (CAG) Amur grayling. Results are shown as mean ± SD (*n* = 5). Values marked with asterisks are significantly different (^**^p < 0.01).

As shown in **Figure 2**, CAT activity in the liver was 9% higher in the captive group than in the wild group (*p* < 0.01). The gut antioxidant parameters of wild and cultured Amur grayling showed highly significant differences (*p* < 0.01). Specifically, CAT activity and GSH content were 64% and 66% higher in the wild group than in the cultured group, respectively, while MDA levels were lower in the wild group.

### 3.4 Expression of immune- and inflammatory-related genes in the liver and gut

**Figure 3:**
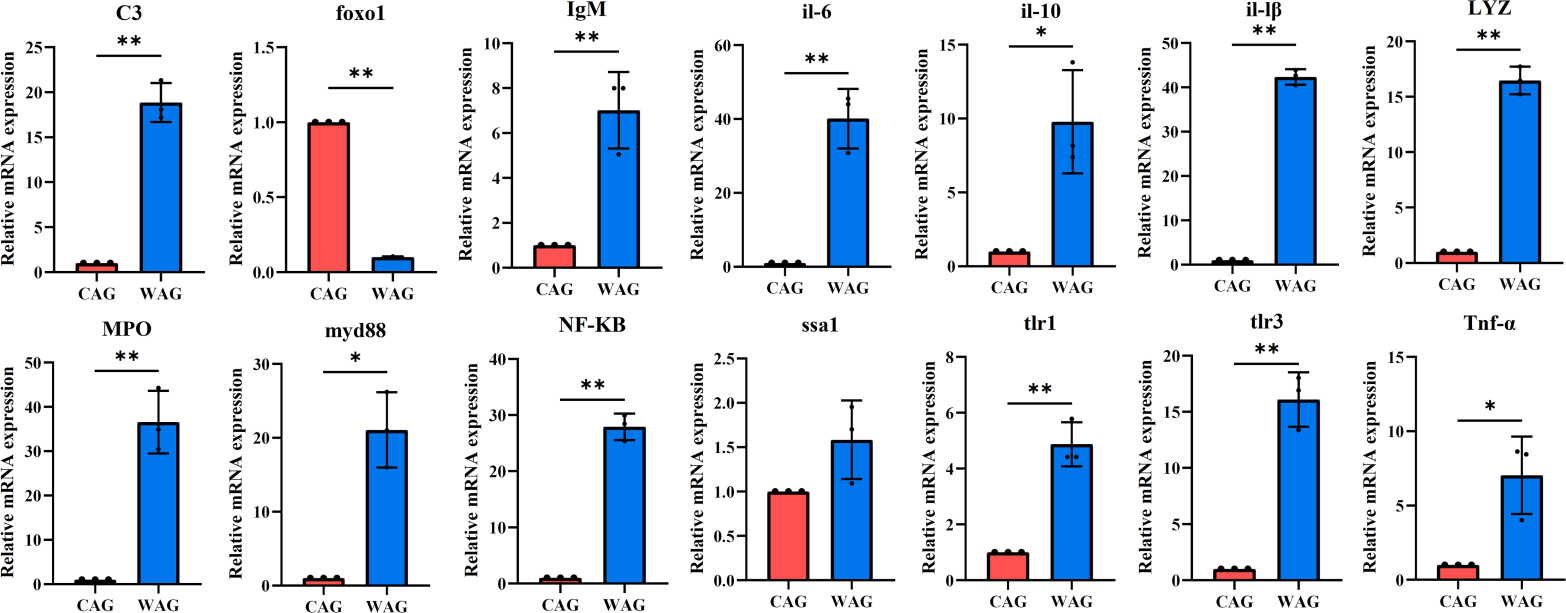
The mRNA expression levels of immune- and inflammatory-related genes in the liver tissue of wild (WAG) and cultured (CAG) Amur grayling. Results are shown as mean ± SD (*n* = 5). Values marked with asterisks are significantly different (^*^p < 0.05; ^**^p < 0.01).

**Figure 4:**
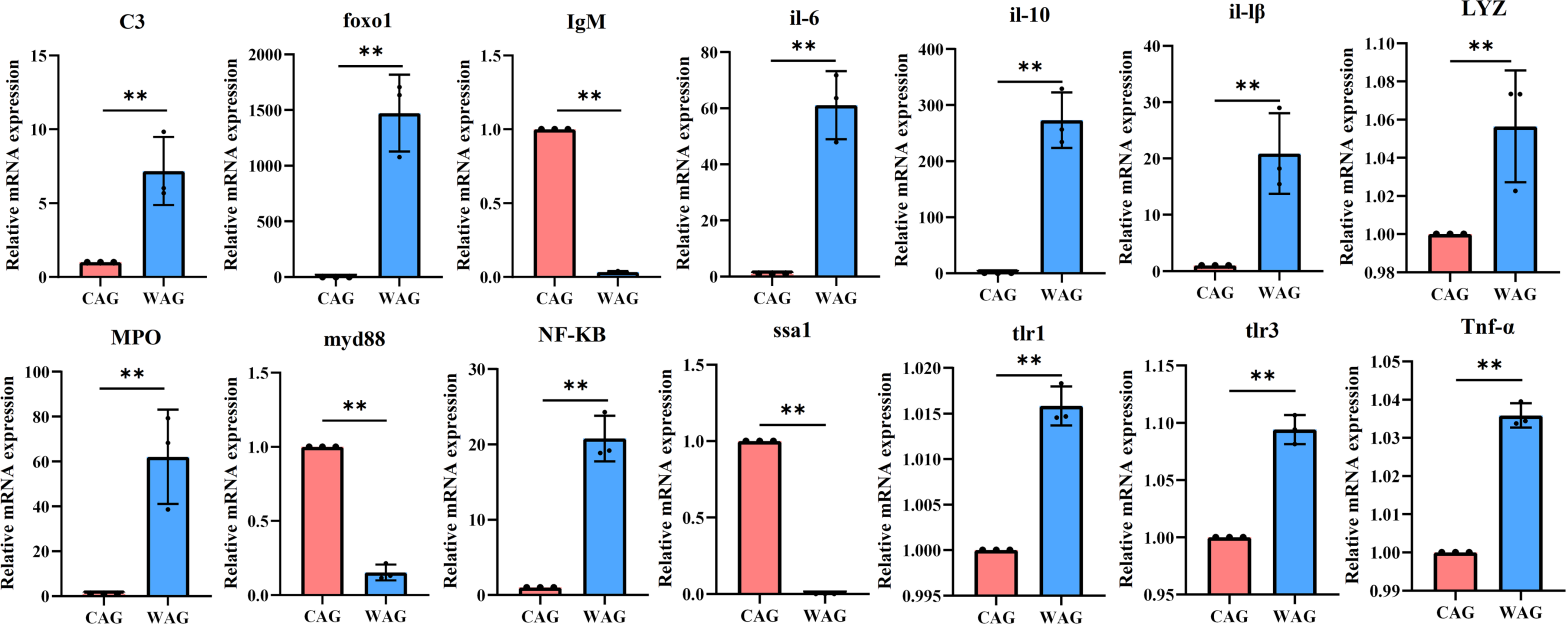
The mRNA expression levels of immune- and inflammatory-related genes in the intestinal tissue of wild (WAG) and cultured (CAG) Amur grayling. Results are shown as mean ± SD (*n* = 5). Values marked with asterisks are significantly different (^*^p < 0.05; ^**^p < 0.01).

In the present study, the mRNA expression levels of immune/inflammatory-related genes in the liver and intestines were measured by qRT-PCR. In the liver, the mRNA expression of *C3*, *IgM*, *il-6*, *il-10*, *il-lβ*, *lyz*, *mpo*, *myd88*, *NF-κB*, *ssa1*, *tlr1*, *tlr3*, and *Tnf-α* was upregulated in the WAG group, while *foxo1* was significantly downregulated (*p* < 0.05) (**Figure 3**). In intestinal tissue, the mRNA levels of *C3*, *foxo1*, *il-6*, *il-10*, *il-lβ*, *tlr1*, *tlr3*, *Tnf-α*, *lyz*, *mpo*, and *NF-кB* increased significantly in the WAG group, whereas the expression of *IgM*, *myd88*, and *ssa1* decreased significantly (*p* < 0.05) (**Figure 4**).

### 3.5 Overview of 16S rRNA gene sequencing and gut microbiota structure

**Figure 5:**
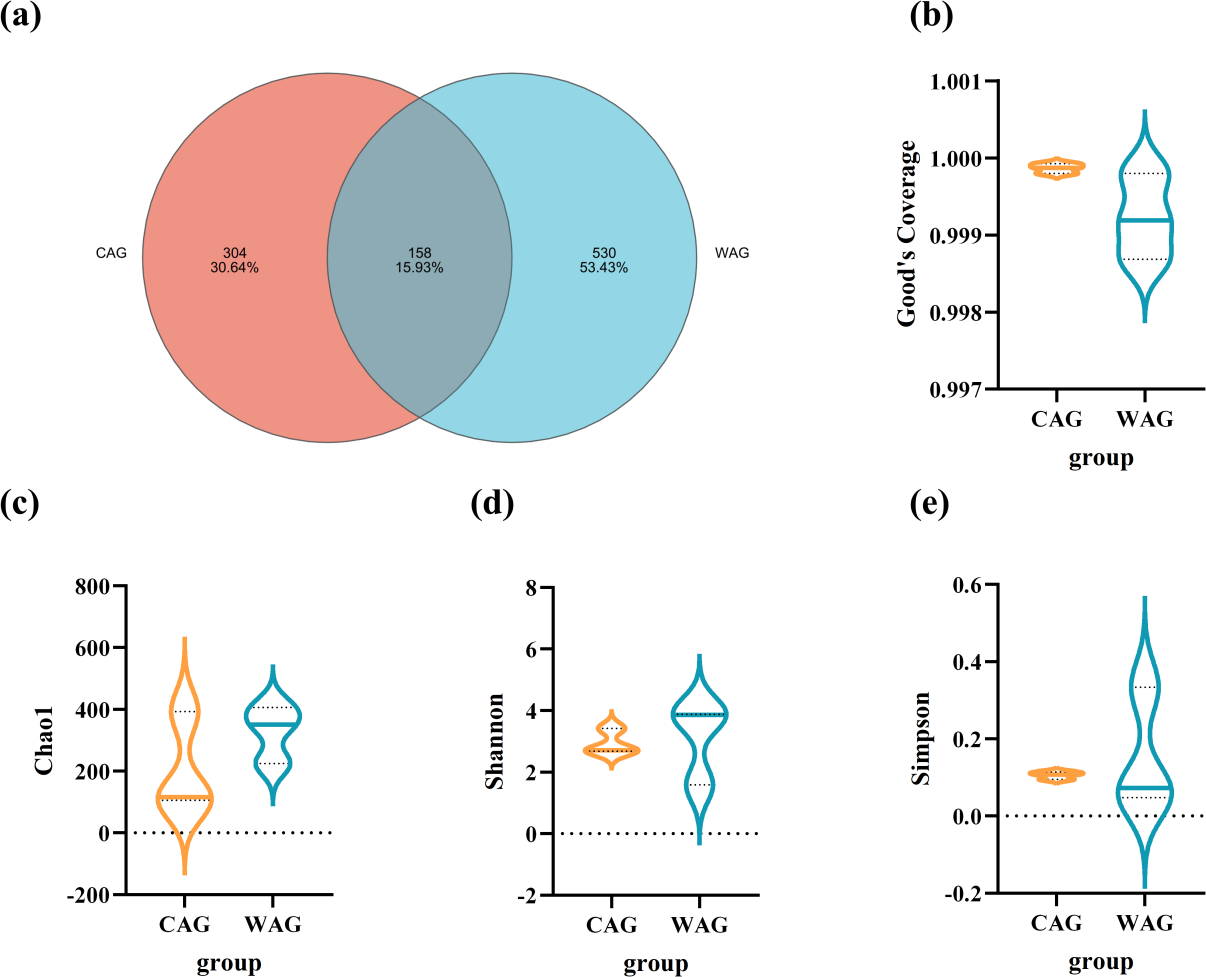
Venn diagram of operating taxonomic units **(a)** and α-diversity analysis **(b–e)** of the intestinal microbiota of wild and cultured Amur grayling. The number of OTUs shared between the wild (WAG) and captive (CAG) groups was 158, with 530 unique OTUs in the wild group and 304 unique OTUs in the captive group.

**Figure 6:**
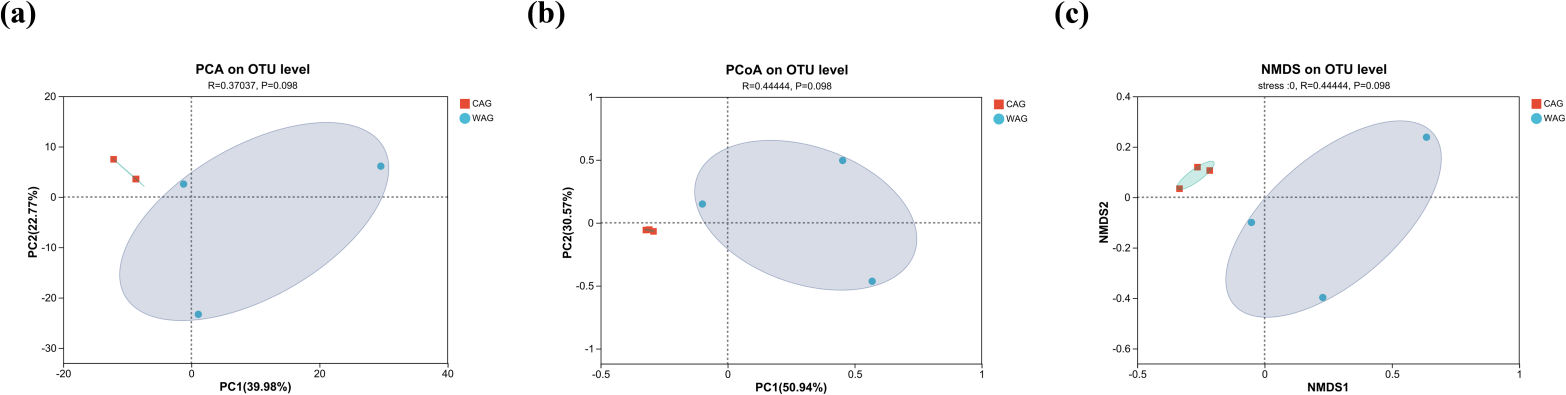
Comparisons of β-diversity in the gut microbiota between wild (WAG) and captive (CAG) Amur grayling. **(a)** Principal component analysis (PCA). **(b)** Principal coordinate analysis (PCoA) **(c)** Nonmetric multidimensional scaling (NMDS).

Using high-throughput 16S rRNA gene sequencing, we analyzed the farm group (CAG; *n* = 3) and WAG (*n* = 3), yielding a total of 404,602 raw reads. The Venn diagram analysis identified 992 OTUs, with 304 OTUs unique to the CAG group and 530 OTUs unique to the WAG group (**Figure 5a**). Good’s coverage confirmed that the sequencing captured up to 99% of the gut microbiota in both captive and wild Amur grayling (**Figure 5b**). The Chao1 index indicated that wild Amur grayling had higher species richness than captive individuals (**Figure 5c**). Additionally, Shannon and Simpson indices showed that the gut microbiota of wild Amur grayling was more diverse, with higher Shannon and lower Simpson values compared with captive Amur grayling (**Figures 5d, e**). These results indicate that fish living in a natural environment exhibit higher species richness and greater species diversity. However, analyses using principal component analysis (PCA), PCoA, and nonmetric multidimensional scaling (NMDS) revealed no statistically significant differences between the gut microbiota of farmed and wild fish (**Figure 6**).

**Figure 7:**
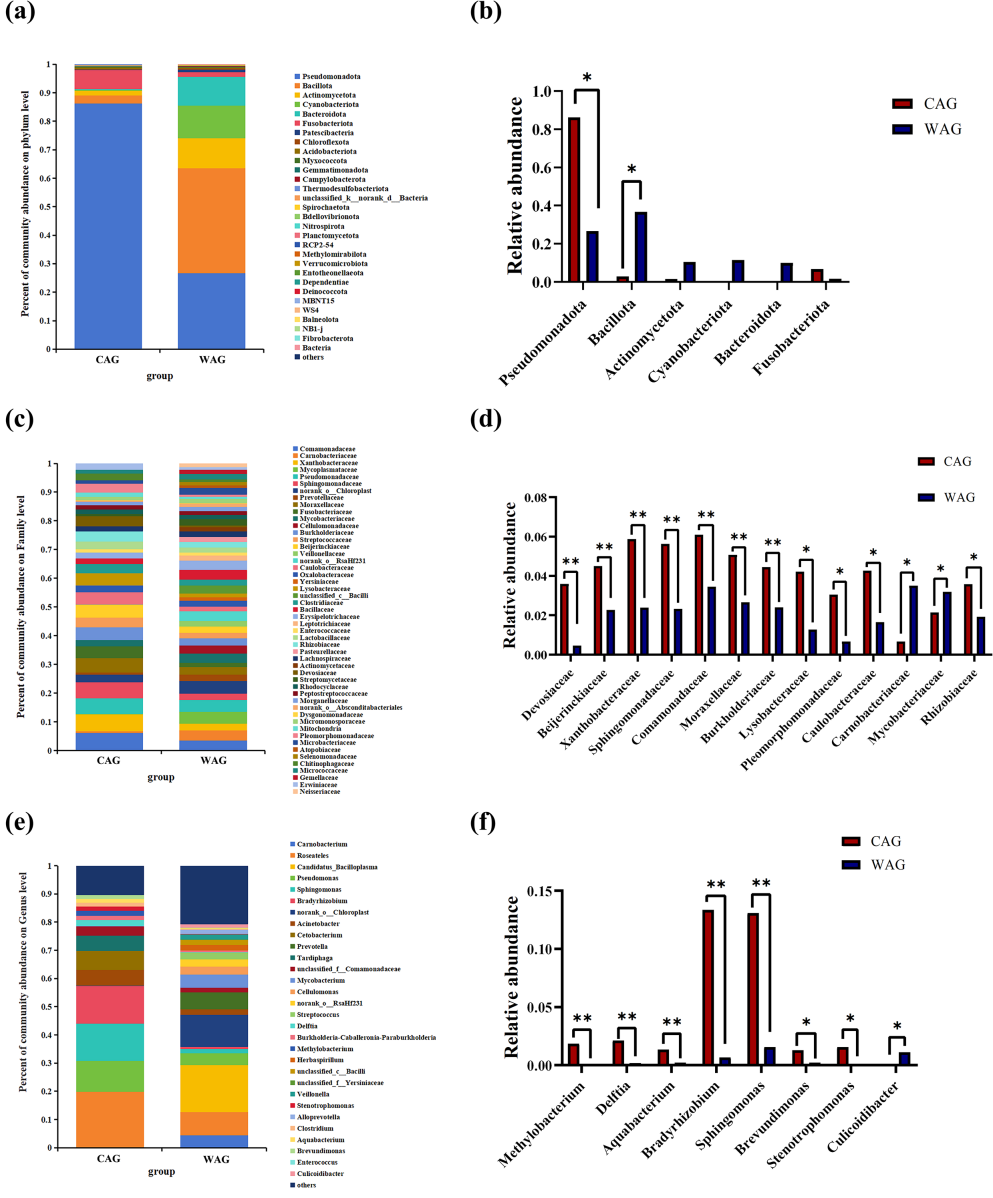
Differences in the relative abundance of intestinal bacterial flora between wild (WAG) and farmed (CAG) Amur grayling. **(a)** Relative abundance of intestinal bacteria at the phylum level. **(b)** Significant bacterial community abundance at the phylum level. Values marked with asterisks are significantly different (^*^p < 0.05). **(c)** Relative abundance of intestinal bacteria at the family level. **(d)** Significant bacterial community abundance at the family level. Values marked with asterisks are significantly different (^*^p < 0.05; ^**^p < 0.01). **(e)** Relative abundance of intestinal bacteria at the genus level. **(f)** Significant bacterial community abundance at the genus level. Values marked with asterisks are significantly different (^*^p < 0.05; ^**^p < 0.01).

To assess gut microbiota differences between wild and aquaculture Amur grayling, samples from Amur Lake were compared with those from an aquaculture facility. At the phylum level, the gut microbiota of farmed Amur grayling was dominated by Pseudomonadota, followed by Fusobacteriota, Bacillota, and Actinomycetota, which together accounted for 97.3% of the community. In contrast, the wild cohort was dominated by Bacillota, followed by Pseudomonadota, Cyanobacteriota, and Actinomycetota, representing 88.7% of the community (**Figure 7a**). Bacillota was significantly more abundant in wild fish than in the farmed group, whereas Pseudomonadota was significantly less abundant in the wild group (*p* < 0.05) (**Figure 7b**). As shown in **Figure 7c**, at the family level, the farmed group was dominated by *Comamonadaceae* (6.10%), followed by Xanthobacteraceae (5.88%). In the wild group, *Mycoplasmataceae* was the most abundant, accounting for 4.2%. Compared with the farmed group, the wild group showed a significant increase in the relative abundance of *Carnobacteriaceae* and *Mycobacteriaceae*, whereas the relative abundances of *Devosiaceae*, *Beijerinckiaceae*, *Xanthobacteraceae*, *Sphingomonadaceae*, *Comamonadaceae*, *Moraxellaceae*, *Burkholderiaceae*, *Lysobacteraceae*, *Pleomorphomonadaceae*, *Caulobacteraceae*, and *Rhizobiaceae* were significantly decreased (*p* < 0.05) (**Figure 7d**). The most abundant genus in the gut microbiota of wild Amur grayling was *Candidatus_Bacilloplasma* (16.6%), followed by *norank_o_Chloroplast* (11.4%), *Roseateles* (8.2%), and *Prevotella* (5.9%) (**Figure 7e**). In captive Amur grayling, *Roseateles* (19.8%) was the dominant genus, followed by *Bradyrhizobium* (13.3%), *Sphingomonas* (13.1%), and *Pseudomonas* (11.1%). Compared with the captive group, the wild group showed a significant increase in the relative abundance of *Culicoidibacter*, whereas the relative abundances of *Methylobacterium*, *Delftia*, *Aquabacterium*, *Bradyrhizobium*, *Sphingomonas*, *Brevundimonas*, and *Stenotrophomonas* were significantly decreased (*p* < 0.05) (**Figure 7f**).

**Figure 8:**
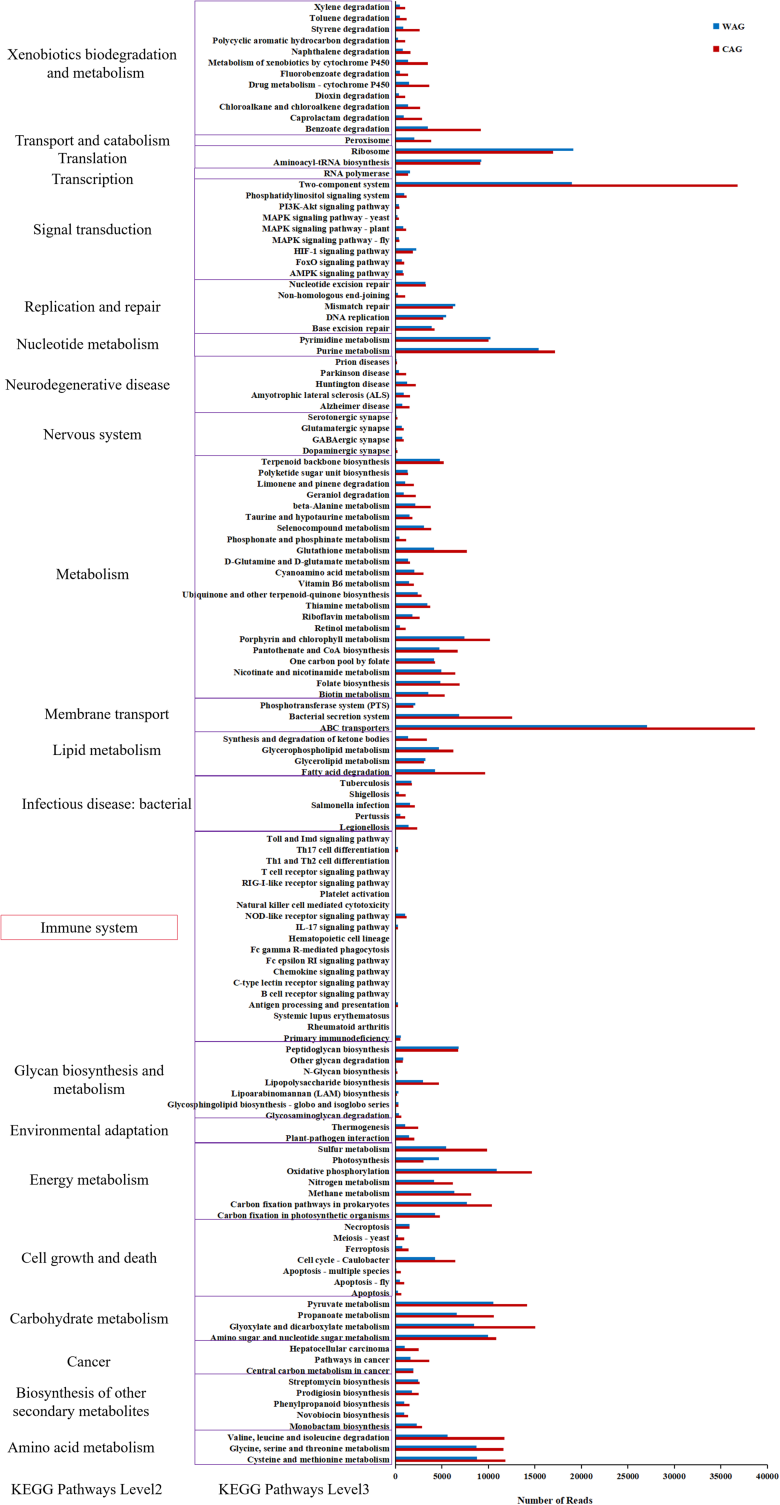
KEGG pathways that are enriched or depleted in the intestinal microbiota of wild (WAG) and farmed (CAG) Amur grayling.

The changes in the presumptive functions of the intestinal microbiota of Amur grayling were examined by predicting the metagenomes using PICRUSt (**Figure 8**). Four functional pathways were more highly abundant in wild Amur grayling, including glycan biosynthesis and metabolism, immune disease, immune system, and translation. In aquaculture Amur grayling, 23 pathways were more highly abundant compared with wild fish, including those related to amino acid metabolism, biosynthesis of other secondary metabolites, carbohydrate metabolism, cell growth and death, and energy metabolism. Overall, these results clearly demonstrate that immune suppression in farmed Amur grayling corresponds to decreased abundances of Bacillota and other potentially beneficial microbes, highlighting the interconnectedness of diet, microbiota, and host immunity.

### 3.6 Correlation between intestinal microbiota and immune- and inflammatory-related indices

**Figure 9:**
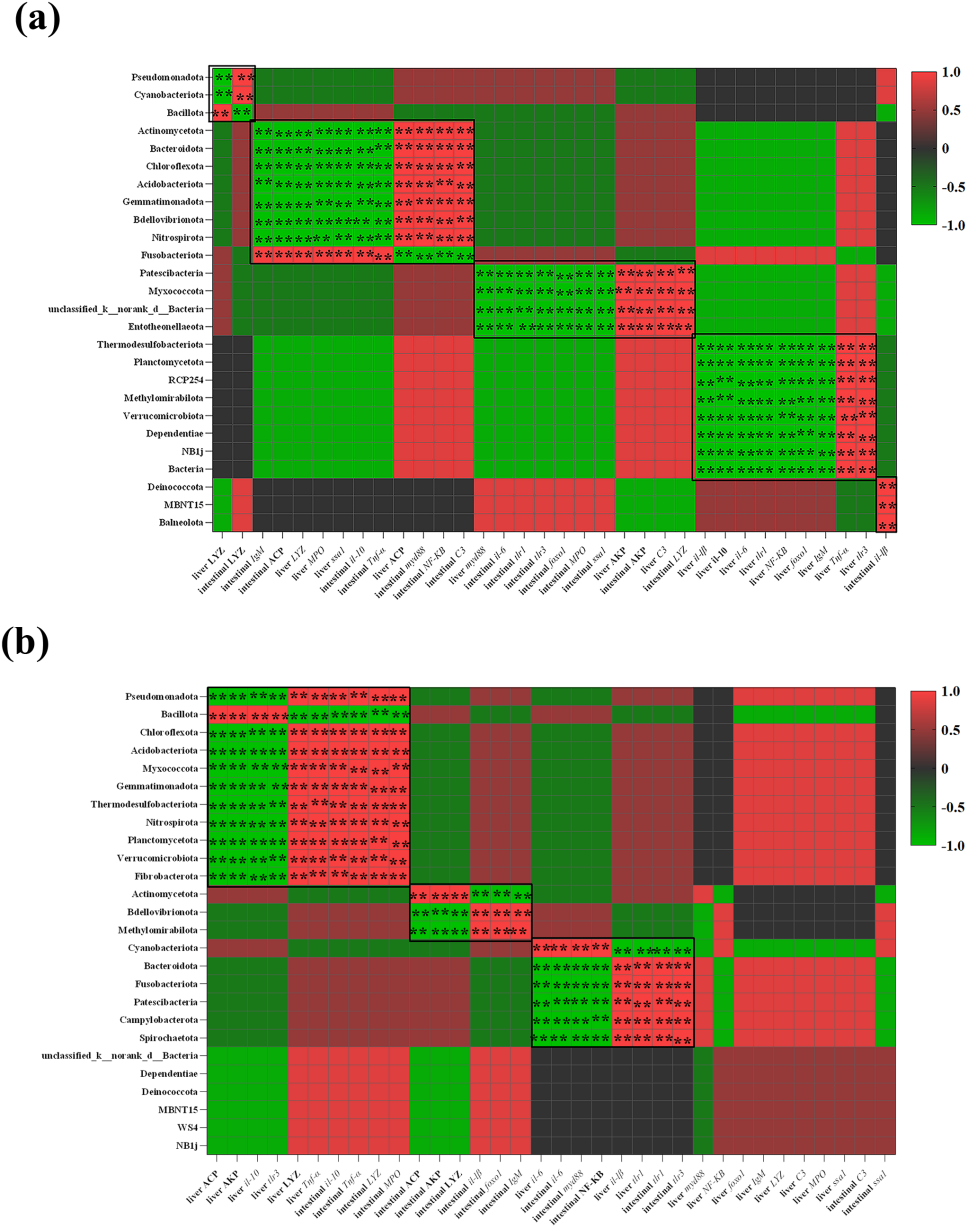
Correlation analyses between the abundance of the gut microbiota and gut and liver immune- and inflammation-related indices. **(a)** Correlation analysis between the gut microbiota at the phylum level of captive Amur grayling and immune- and inflammation-related indices. **(b)** Correlation analysis between the gut microbiota at the phylum level of wild Amur grayling and immune- and inflammation-related indices (^**^p < 0.01). Red represents a positive correlation, while green indicates a negative correlation.

Spearman correlation coefficient was used to test the relationship between the abundance of intestinal microbiota at the phylum level and immune- and inflammatory-related indices (**Figure 9**). Significant correlations were observed between intestinal and liver immune-related indicators (i.e., liver ACP, liver AKP, liver LYZ, liver *il-10*, liver *Tnf-α*, liver *tlr3*, intestinal *il-10*, intestinal *Tnf-α*, intestinal *lyz*, intestinal *mpo*) and the gut microbiota (i.e., Pseudomonadota, Bacillota, Chloroflexota, Acidobacteriota, Thermodesulfobacteriota, Planctomycetota, Verrucomicrobiota, Fibrobacterota) in the WAG group, as shown in the heatmap. Pseudomonadota, Chloroflexota, Thermodesulfobacteriota, Planctomycetota, Verrucomicrobiota, and Fibrobacterota were positively correlated with LYZ and *Tnf-α* in the liver and with *il-10*, *Tnf-α*, *lyz*, and *mpo* in the intestine (*R* > 0.5, *p* < 0.01), but negatively correlated with ACP, AKP, *il-10*, and *tlr3* in the liver (*R* ≤ 0.5, *p* < 0.01). Bacillota was strongly positively correlated with liver ACP, liver AKP, liver *il-10*, and liver *tlr3* (*R* > 0.5, *p* < 0.01), and negatively correlated with liver *lyz*, liver *Tnf-α*, intestinal *il-10*, intestinal *Tnf-α*, intestinal *lyz*, and intestinal *mpo* (*R* ≤ 0.5, *p* < 0.01). Spearman correlation was performed between immune parameters and phylum-level intestinal microorganisms in the CAG group. The relative abundance of Actinomycetota, Bacteroidota, Chloroflexota, Acidobacteriota, Bdellovibrionota, and Nitrospirota was significantly positively correlated with liver ACP and intestinal *myd88*, *NF-κB*, and *C3* mRNA levels (*R* > 0.5, *p* < 0.01). However, their relative abundance was significantly negatively correlated with intestinal ACP, *il-10*, *Tnf-α*, and *IgM*, as well as with liver *lyz*, *mpo*, and *ssa1* (*R* ≤ 0.5, *p* < 0.01), whereas Fusobacteriota showed the opposite correlation. The abundance of Patescibacteria, Myxococcota, unclassified_k_norank_d_Bacteria, and Entotheonellaeota was positively correlated with immune indices (AKP, liver *C3*, intestinal *lyz*) and inversely correlated with genes (liver *myd88*, intestinal *il-6*, *tlr1*, *tlr3*, *foxo1*, *mpo*, *ssa1*) that enhance immunity.

## 4 Discussion

As an important digestive organ, the intestine absorbs nutrients from the diet and participates in various immune responses of the body (22). Due to the different feed types, foraging habits, and habitats between wild and captive cold-water fish, it is anticipated that their gut microbiota communities will differ (23). Our main observation is the significant difference in the microbiota depending on the origin of the fish (wild or farmed) and the immune functions of wild and farmed Amur grayling, as well as exploring the interactions between the host immunity and intestinal microorganisms, which is very meaningful for maintaining fish health.

### 4.1 Comparative analysis of the immune system between wild and captive Amur grayling

The fish immune system is crucial for host defense and is considered an important marker for disease prevention and for maintaining fish health (24). In our trial, a series of immune-related indicators in the liver and gut tissues of wild and farmed Amur grayling were measured. Among them, significantly higher AKP and LYZ activities were found in the liver and gut tissues of wild Amur grayling. AKP and LYZ are important components of innate immunity and play a vital role in mediating host protection against bacterial invasion (25, 26). This study indicated the superior innate immune performance of wild Amur grayling.

The intestinal barrier is composed of biological, chemical, mechanical, and immune barriers, which can protect the host from pathogen invasion (27). Chemical barriers include a series of chemical substances such as complement proteins, lysozyme, and intestinal antimicrobial peptides in the outer mucus layer of intestinal epithelial cells (28, 29). In this study, the expression levels of genes that encode *C3*, *lyz*, and antibacterial enzymes such as *tlr1* and *tlr3* were significantly elevated in wild Amur grayling, implying superior intestinal chemical barrier function in wild Amur grayling. Inflammation is a protective biological response of the body to external injury and is mediated by several cytokines (30). In fish, the immune barrier in the intestine largely depends on their immune response (31). Lu et al. (32) have shown that a successful host immune response is generally the result of the actions of both pro- and anti-inflammatory cytokines, which clear pathogens and reduce tissue damage. The cytokines *Tnf-α*, *il-lβ*, and *il-6* are considered proinflammatory cytokines (33). However, the anti-inflammatory cytokine *il-10* is an immunoregulatory molecule that regulates the activity of proinflammatory cytokines (34). Currently, few studies have reported on the immunological barrier in wild and cultured Amur grayling. The present study revealed the upregulated mRNA expression trends of pro-inflammatory genes (*Tnf-α*, *il-lβ*, and *il-6*) and anti-inflammatory genes (*il-10*) in the liver and intestine of wild Amur grayling. TLRs can activate *NF-κB* by mediating *myd88* to enhance the formation of proinflammatory factors (35). The wild individuals had higher relative mRNA expression levels of *myd88* and *NF-κB* in the liver than the cultured group. The results of this study indicated that Amur grayling exhibit enhanced antimicrobial effects and immune function in the wild. In addition, IgM produced by B lymphocytes participates in specific immune responses and is an important indicator for measuring immune response capability (36). In this study, significantly higher mRNA expression of *IgM* in the liver was observed in wild Amur grayling, whereas the expression of *IgM* decreased significantly in the gut, implying that the farm and wild environments activate different immune-related genes to mediate various immune responses in a tissue-specific manner.

In normal metabolism, the dynamic balance of redox maintains a stable state. When fish are subjected to internal and external stress, reactive oxygen species are produced, and the balance of the antioxidant system in the body is impaired, ultimately leading to a reduction in intestinal health, immunity, and growth performance (37, 38). SOD, CAT, GSH, and MDA are important indicators of antioxidant function. SOD, CAT, and GSH can scavenge free radicals to reduce damage to the intestinal mucosa caused by oxidative stress, playing important roles in intestinal defense and repair (39, 40), while MDA content represents the degree of lipid peroxidation and indirectly reflects the extent of cellular damage (41). In the gut, the activities of antioxidant enzymes in wild Amur grayling were significantly higher, while the MDA levels in the liver and gut were lower compared with those in cultured individuals, indicating that the antioxidant capacity of wild Amur grayling is stronger than that of cultured ones.

### 4.2 Differences in the composition of the gut microbiota between wild and captive Amur grayling

Intestinal microbiota constitute a complex and diverse ecosystem comprising many microorganisms (42). The gut microbiome is crucial for the health and survival of fish (43). In this study, the dominant phyla in wild and captive groups were Bacillota and Pseudomonadota, respectively. This result was similar to a previous comparative study of the gut microbiota in wild and farmed Malaysian Mahseer (*Tor tambroides*) by Tan et al. (2019). Similarly, Pseudomonadota is also regarded as the dominant phylum in many captive fish species, such as rainbow trout (*Oncorhynchus mykiss*) and Atlantic salmon (*Salmo salar*) (44, 45). Moreover, it has been demonstrated that an increased abundance of Pseudomonadota is a potential diagnostic signature of metabolic and immune dysbiosis (46). Zhao et al. (47) reported that the abundance of Pseudomonadota increased after immune suppression. Therefore, we hypothesize that Pseudomonadota could inhibit the immune responses and decrease the expression of hepatic and intestinal immune-related genes in farmed Amur grayling.

Studies have shown that Bacillota could provide nutrients and energy substances to epithelial and gastrointestinal cells, which can improve mucus production, act as an anticancer and anti-inflammatory agent, and maintain intestinal barrier integrity (48). In cultured Amur grayling fish, the significantly increased abundance of Pseudomonadota and decreased abundance of Bacillota caused immune suppression, as indicated by the significantly downregulated relative expression of *lyz*, *NF-κB*, and *il-10*. Similar findings have been reported in cultured Japanese seabass (*Lateolabrax japonicus*) exposed to high dietary soybean meal levels (49). Dysbiosis of the intestinal microbiota could lead to disorders of the intestinal immune system and disease occurrence in farmed Amur grayling. In addition, Bacillota showed a greater abundance in the wild group than in the farm group. It was speculated that the gut health of wild Amur grayling was superior to that of captive Amur grayling. Ammonia is the inorganic nitrogen source for various Cyanobacteriota taxa (50). Compared with the farm group, Cyanobacteriota increased in the wild group, which may have been caused by the reproduction of Cyanobacteriota in the water and subsequent ingestion by the Amur grayling. Similar results have been reported for oysters (*Crassostrea gigas*) (51). Therefore, it is hypothesized that the ammonia content in wild habitats might be higher than that in aquaculture water, which could promote an increased stress response to ammonia exposure in wild Amur grayling.

The use of large amounts of organic and inorganic fertilizers, fecal waste generation, and low-quality feed conversion induces pollution in captive water (52). Delftia can degrade and transform organic and inorganic pollutants in the aquatic environment (53, 54). Thus, Delftia showed a higher relative abundance in the captive group. Studies have shown that frequent exposure of cultured fish to various pollutants leads to a decline in intestinal health and immunity (55). We hypothesized that this results in reduced immune function in cultured Amur grayling. High-carbohydrate and high-lipid diets have been widely used in aquaculture practices, but they can also cause excessive lipid accumulation in fish liver (56). In contrast, wild Amur grayling mainly consume small fish and zoobenthos with a high protein content. As observed in many animals, diet is a key factor influencing the gut microbiota (57). Thus, the gut microbiota in captive Amur grayling showed higher carbohydrate metabolism levels. In addition, increases in “carbohydrate metabolism” and “lipid metabolism” were observed. The pronounced disparity in dietary composition between farmed and wild Amur grayling profoundly shapes their respective gut microbial communities. We therefore encourage optimal wilderness training before artificial stock enhancement to adjust the gut microbial community of fish species and improve their adaptability to wild populations in reintroduction projects.

### 4.3 Interactions between host immunity and intestinal microorganisms in captive and wild Amur grayling

Interactions between the host and intestinal microbiota form the basis for the development of immunity (58). In our study, Actinomycetota, Bacteroidota, and Bdellovibrionota were positively correlated with the immune parameter (liver ACP) and intestinal immune-related genes (*myd88*, *NF-κB*, and *C3*) in captive Amur grayling. According to Jakubiec-Krzesniak et al. (59), Actinomycetota can secrete secondary metabolites that could inhibit pathogenic bacteria. Bdellovibrionota can disrupt the colonization of the *Shigella* genus, thereby enhancing the survival of zebrafish (*Danio rerio*) (60). Bacteroidota can improve the decomposition of nutrients and digestibility of the host, and their abundance is closely related to maintaining the intestinal environmental balance in animals (61). Therefore, the decreased abundances of Actinomycetota, Bdellovibrionota, and Bacteroidota in cultivated Amur grayling allowed undigested nutrients and harmful substances to enter into liver through the hepatic portal vein, thereby inducing a liver immune response. In addition, Actinomycetota, Bdellovibrionota, and Bacteroidota were considered important factors contributing to the increased survival rate of Amur grayling in the wild environment after artificial enhancement and release. Fusobacteriota have protective effects on fish and can produce butyrate and vitamin B12, which are involved in the immune-regulatory processes in the body (62). In our study, Fusobacteria were positively associated with intestinal immune response cytokine *IgM*, and both the relative abundance of Fusobacteria and the expression level of IgM increased in the captive group. It has been demonstrated that Fusobacteria are a gut microbiota biomarker closely related to the immune response in captive Amur grayling.

The gut microbiota regulates immunity via the gut axis. Planctomycetota influence complex chemical metabolism. In addition, Verrucomicrobiota benefit fish gut health by enhancing immune responses, regulating microbial communities, and promoting mucosal repair (63). Fibrobacterota are small but important phyla, mainly reported as cellulose-degrading bacteria (64). In addition, Thermodesulfobacteriota, Planctomycetota, Verrucomicrobiota, and Fibrobacterota were positively associated with inflammatory factors (hepatic LYZ and *Tnf-α*, intestinal *il-10*, *Tnf-α*, *lyz*, and *mpo*) but negatively correlated with liver immune- and inflammatory-related parameters (ACP, AKP, *il-10*, and *tlr3*) in the intestine tissue of wild Amur grayling. Bacillota showed the opposite correlation. The results indicated that Amur grayling immune responses can be linked to the abundance levels of Bacillota, Planctomycetota, Verrucomicrobiota, and Fibrobacterota. Based on the above studies, we postulate that intestinal microbes in Amur grayling may modulate host immunity by enhancing the expression of immune-related genes (*il-10*, *Tnf-α*, *lyz*, and *mpo*). Further research is required to understand how host–microbiome interactions coevolve and adapt, as well as the specific roles of certain microorganisms in nutrient processing and immune regulation. Studying the gut microbiomes of wild fish populations is important for understanding their natural microbial communities and ecological functions, in addition to research focused on aquaculture or laboratory fish.

### 4.4 Limitations

Firstly, we acknowledge that our experimental groups comprised *n* = 15 fish per treatment, which limits the statistical power to detect subtle microbiota shifts. Secondly, all farmed samples were collected from the Bohai Cold Water Fish Experimental Station of the Heilongjiang Institute of Fisheries Research; thus, the findings may not generalize to Amur grayling in other geographic regions or rearing systems. Thirdly, all wild samples were captured in the Huma River, from the upper Amur River, in autumn. The diet of Amur grayling varies with the season; thus, the results of comparisons of gut microbiota between wild and farmed Amur grayling may differ across seasons.

### 4.5 Future directions

Firstly, we outline plans for repeated sampling from the same individuals across an entire production cycle to capture temporal trajectories of the gut microbiota. Secondly, we propose controlled feeding experiments with candidate probiotic strains (e.g., Bacillota isolated from wild conspecifics) to test whether targeted microbial interventions can restore immune-relevant taxa in farmed fish.

## 5 Conclusion

In conclusion, this study revealed the differences in the composition of the intestinal microbiota and immune response between wild origin and aquacultured Amur grayling, while the liver–gut–microbiota axis may play an essential role in the underlying mechanisms. Moreover, Actinomycetota, Bdellovibrionota, Bacteroidota, and Fusobacteriota might provide important functions for the increased survival rate of Amur grayling in the wild environment after artificial enhancement and release. Future research could focus on isolating those bacteria that may be used as potential probiotics. The results of this study provide a promising direction for the healthy aquaculture and species reintroduction programs of Amur grayling and offer a theoretical basis for its conservation.

## Data Availability

The datasets presented in this study can be found in online repositories. The names of the repository/repositories and accession number(s) can be found below: https://www.ncbi.nlm.nih.gov/, PRJNA1309249.
