# Supplementary material for: Characteristics and differences in immune response capacity and gut microbiome between wild and captive Amur grayling (Thymallus grubii): New insights into endangered fish conservation
Source: Front Immunol. 2025 Nov 19;16:1654437. doi: 10.3389/fimmu.2025.1654437 (PMC12672233; doi:10.3389/fimmu.2025.1654437)
Supplement: Supplementary file 1 [file DataSheet1.docx]

**Tab. S1 The nutrient content of feed for Amur grayling**

| Nutrient content indicators (g/100g) | |
| --- | --- |
| Crude protein | 43.4 |
| Crude fat | 20 |
| Crude fiber | 3 |
| Crude ash | 9 |
| Water content | 8.5 |
| Total Phosphorus | 12 |
| Ca | 0.8 |
| Amino acid | 3.3 |

**Tab. S2 Water quality parameters of wild and farmed environments**

|  | Wild group | Farmed group |
| --- | --- | --- |
| Water temperature | 9.1±0.64℃ | 9℃ |
| Dissolved oxygen (DO) | 7.7 ± 1.2 mg/L | 7.2 ± 0.3 mg/L |
| pH value | 6.5 ± 1.06 | 6.8 ± 0.12 |

**Tab. S3 Age information of all experiment fish**

| **Groups** | **Age (years)** |
| --- | --- |
| wild | 2^+^ |
| farmed | 2^+^ |
